# A Simple, interpretable method to identify surprising topic shifts in scientific fields

**DOI:** 10.3389/frma.2022.1001754

**Published:** 2022-10-12

**Authors:** Lu Cheng, Jacob G. Foster, Harlin Lee

**Affiliations:** ^1^Department of Mathematics, University of California, Los Angeles, Los Angeles, CA, United States; ^2^Department of Sociology, University of California, Los Angeles, Los Angeles, CA, United States

**Keywords:** science of science, cognitive science, topic modeling, topic matching, matrix factorization, entropy

## Abstract

This paper proposes a text-mining framework to systematically identify vanishing or newly formed topics in highly interdisciplinary and diverse fields like cognitive science. We apply topic modeling via non-negative matrix factorization to cognitive science publications before and after 2012; this allows us to study how the field has changed since the revival of neural networks in the neighboring field of AI/ML. Our proposed method represents the two distinct sets of topics in an interpretable, common vector space, and uses an entropy-based measure to quantify topical shifts. Case studies on vanishing (e.g., connectionist/symbolic AI debate) and newly emerged (e.g., art and technology) topics are presented. Our framework can be applied to any field or any historical event considered to mark a major shift in thought. Such findings can help lead to more efficient and impactful scientific discoveries.

## 1. Introduction

The science of science uses quantitative and computational approaches to examine science itself. It measures and models the interactions between scientific agents and entities in order to understand scientific practices, improve scientific processes, and promote scientific innovation (Evans and Foster, 2011; Fortunato et al., 2018; Lee et al., 2021; Portenoy et al., 2021). An important research topic in the science of science studies how fields form, evolve, and disappear over time. This is particularly challenging — but also rewarding—for complex, highly interdisciplinary subjects like cognitive science. For example, Núñez et al. (2019) have argued that although cognitive science was designed to integrate six disciplines (psychology, linguistics, artificial intelligence, anthropology, philosophy and neuroscience), it has failed to transition into a coherent interdisciplinary field; instead, it is dominated by cognitive psychology.

This paper presents a novel text-mining framework to systematically identify vanishing or newly formed topics in a highly interdisciplinary and diverse field like cognitive science. In contrast to the bibliometric and socio-institutional (i.e., *contextual*) analysis done in Núñez et al. (2019), we employ topic modeling on the scientific literature *content* to examine the change in topic compositions (Evans and Foster, 2011). Bibliographic information (i.e., context) and text information (i.e., content) provide different lenses on the structure and evolution of scientific fields (Evans and Foster, 2011; Vilhena et al., 2014). Topics extracted from text have some advantages. Themes can be easier to interpret, relying less on human labels or socio-institutional structures like journal names. Text also allows us to see connections between fields that are not visible at the level of citation, because socio-institutional factors may lead authors to point to distinct literatures when discussing substantively similar material (Cheng et al., 2021).

We build on a long-standing tradition of using citations and text to identify topics and their changes over time. Tools like CiteSpace (Chen, 2016) have long enabled the extraction of dynamic clusters from bibliographic information (including automatic cluster labeling), and flow-based, dynamic community detection methods (Rosvall and Bergstrom, 2010) have identified the emergence of new interdisciplinary fields like neuroscience. On the text side, dynamic topic models (Blei and Lafferty, 2006) can trace the changing content of topics over time, while recent extensions (Gerow et al., 2018) can quantify the influence of past documents on the content of future topics (while also providing evidence for the complex relationship between citations and textual influence).

Our approach most closely follows the text-based, topic modeling tradition. Using the cognitive science publications dataset curated in Cheng et al. (2021), we apply topic modeling separately to abstracts published before and after the seminal AlexNet paper appeared in the adjacent AI/ML literature (Krizhevsky et al., 2012). We propose a method to 1) intuitively represent the two distinct sets of topics in a common mathematical space and 2) use a novel entropy-based measure to quantify changes in topics. Brief case studies on vanished (e.g., symbolic AI) and newly emerged (e.g., art and technology) topics, along with a quick comparison to a landmark publication internal to cognitive science (Rumelhart et al., 1986) illustrate the promise of our approach for more detailed investigations. The chief value of our approach lies in its use of simple, well-understood, and efficient building blocks. This makes it easy to deploy, interpret, and improve (e.g., by swapping out different topic modeling approaches).

While this paper focuses on how *cognitive science* has changed since *the revival of artificial neural networks in 2012*, we emphasize that this literature-based discovery framework can be applied to any scientific field and any historical event of interest. Scholars can identify potential change-points using a range of qualitative evidence, including histories of the field, review articles, interviews with domain experts, or their own domain knowledge. These tools can help researchers make more thoughtful decisions on research problems, identifying fields that may have been prematurely abandoned or unexpected genealogies of newly emerged fields. Such refined understanding of how fields grow and change will result in more efficient allocation of scientific effort and more powerful scientific innovations. Our approach can also be a valuable tool for scholars interested in the history and evolution of scientific disciplines or other discursive fields.

## 2. Method

First, we describe our general framework for identifying topics that have dissipated or materialized after a historical event in complex and diverse scientific fields. This combines a series of natural language processing (NLP) methods and well-established mathematical procedures (e.g., cosine similarity, entropy) through a new lens to detect topic trends from text data.

1. Identify a historical event of interest *t*, e.g., publication of a seminal paper.
2. Apply topic modeling to publications before and after *t*, then assign each paper to its most represented topic. Repeat the process with the entire dataset to get *reference* topic clusters.
3. Represent each topic as a vector, whose elements count the number of papers shared with the reference clusters. This represents topics from non-overlapping time periods in a common mathematical space that is easy to understand and robust to vocabulary changes.
4. Compute a cosine similarity matrix for every pair of topic clusters from before and after *t*. This quantifies topic similarities without relying on human labeling or keyword matching.
5. Calculate the entropy of each row and each column of the similarity matrix to measure how much a topic from before *t* can be “explained away” by a single topic from after *t*, and vice versa. For example, maximum entropy will be achieved when the pre-*t* topic is equally similar to all post-*t* topic vectors, or when the post-*t* topic is equally similar to all pre-*t* topics. This captures an element of surprise and therefore a potential topical shift.
6. Investigate content (keywords) and context (authors, affiliations, journals) of topics with high entropy.

The following sections explain the process in further detail, as applied to studying how content discussed in cognitive science papers has shifted since the publication of Krizhevsky et al. (2012).

### 2.1. Data acquisition and split

We use the publications dataset from Cheng et al. (2021). Initially, a total of 258,039 papers tagged *cognitive science* were accessed from the Microsoft Academic Graph (Sinha et al., 2015) on July 2nd 2021. From the 200,000 papers with the highest probabilities of being “important” as determined by Shen et al. (2018), the authors removed papers published prior to 1950 in order to limit the scope to the modern notion of cognitive science (Núñez et al., 2019). They then kept only the papers that contain references, and whose abstracts are between 30 and 500 words long. Many short abstracts are actually titles and publication information, while long abstracts tend to be tables of contents or the text of the entire first page of the paper. Finally, after removing all papers with duplicate abstracts, we have a dataset of 59,384 papers for further analysis.

Since we are interested in learning about how scientific fields have changed and developed through time, we select a checkpoint in history for *t* and look at how topics have changed before and after *t*. We eventually select the year *t* = 2012, in which the seminal paper by Krizhevsky et al. (2012) revived neural networks, forever changing the landscape of artificial intelligence and (we hypothesize) the adjacent field of cognitive science. We might expect some substantial impact, because many of the ideas behind deep learning were pioneered within cognitive science (Rumelhart et al., 1986). 2012 is roughly a half-way point and gives us similar number of papers for before and after (26,859 and 32,535). Mathematically, we denote the resulting sets—all papers, papers before 2012 and papers after 2012—as *c*, *c*^−^, and *c*^+^ respectively.

### 2.2. Topic modeling and paper assignments

The text preprocessing step follows Cheng et al. (2021). We first lemmatize the abstracts; remove numbers and punctuation; remove English stop words, and stop words specific to abstracts (e.g., “et al,” “this paper”); use Gensim bigram model to turn frequent word combinations into bigrams and trigrams, and filter out words that are too frequent (in more than 80% of abstracts) or too infrequent (in less than 0.05% of abstracts). Finally, we construct the data matrix using term frequency-inverse document frequency (tf-idf) vectorization (Rajaraman and Ullman, 2011). This yields three word-by-abstract matrices *X*∈ℝ^9106 × 59384^, *X*^−^∈ℝ^8852 × 26859^, *X*^+^∈ℝ^8576 × 32525^ from the abstracts in *c, c*^−^, *c*^+^ respectively.

Non-Negative Matrix Factorization (NMF) (Lee and Seung, 1999; Xu et al., 2003; Gillis, 2014) is used to detect latent semantic topics and assign papers to topics; we chose NMF because it is a simple and fast method experimentally shown to generate more stable and interpretable topics, especially on shorter text, compared to other topic model such as LDA (Chen et al., 2019; Egger and Yu, 2022). NMF approximates *X*≈*WH* by


(1) $$\begin{array}{l} \underset{W\geq 0,H\geq 0}{\inf}\;||X-WH|{|}_{\text{F}}^{2}, \end{array}$$

where the dictionary matrix *W* and the coding matrix *H* are two low-rank (rank *n*) matrices with non-negative elements. The *i*th column of *W* gives the weights of the words in the *i*th topic, while the *j*th column of *H* gives the weights of the topics in the *j*th abstract. We assign each paper to its most weighted topic based on *H*, and describe each topic with its top weighted words from *W*. Each paper is assigned to exactly one topic. The topics are ordered based on the number of assigned papers, which gives us paper groups $c_{i},c_{i}^{-},$ and $c_{i}^{+}$ for *i* = 1, …, *n*. For example, $c_{1}^{-}$ is the topic cluster with the highest number of papers in the corpus before 2012. Note that $\cup_{i=1}^{n}c_{i}=c,\cup_{i=1}^{n}c_{i}^{-}=c^{-},\cup_{i=1}^{n}c_{i}^{+}=c^{+}$. After exploring *n* = 10, 50, 100, 200, 500, we chose *n* = 50, as it gave us clear and diverse topics that are neither too broad or too specific by inspection.

### 2.3. Topic representation and entropy-based measurement of change

In order to compare topics before and after 2012, we represent clusters ${\left\{c_{i}^{-}\right\}}_{i=1}^{n}$ and ${\left\{c_{j}^{+}\right\}}_{j=1}^{n}$ in the same vector space—as a distribution on *c*_1_, *c*_2_, …, *c*_*n*_. This allows us to compute similarities and dis-similarities, and finally the entropy in a way that is robust to semantic changes and human biases; topics are represented by their usage in modeling specific texts in the corpus. Let $T_{i}^{-}\in {\mathbb{R}}^{n}$ and $T_{j}^{+}\in {\mathbb{R}}^{n}$ be the vectors describing $c_{i}^{-}$ and $c_{j}^{+}$, respectively, in dimensions of *c*_1_, *c*_2_, …, *c*_*n*_. The *k*-th entry of $T_{i}^{-}$ (or $T_{j}^{+}$) is the number of papers that are both contained in *c*_*k*_ and $c_{i}^{-}$ (or $c_{j}^{+}$).

We denote the cosine similarity (Singhal, 2001) between $T_{i}^{-}$ and $T_{j}^{+}$ as *S*_*ij*_ and assemble the matrix $S=\left[S_{ij}\right]\in {\mathbb{R}}^{n\times n}$ such that the *i*th row of *S* describes how similar $c_{i}^{-}$ is to the clusters ${\left\{c_{k}^{+}\right\}}_{k=1}^{n}$, and the *j*th column represents $c_{j}^{+}$ in terms of its similarity to ${\left\{c_{k}^{-}\right\}}_{k=1}^{n}$. Since every element of *S* is non-negative, the rows and columns of *S* can be treated as a probability distribution once they are normalized to sum to one. Therefore, we compute entropy for each row and column of the matrix *S* to get $H_{1}^{-},H_{2}^{-},\ldots ,H_{n}^{-}$, and $H_{1}^{+},H_{2}^{+},\ldots ,H_{n}^{+}$^1^. By formula,


(2) $$\begin{array}{l} H_{i}^{-}=-\overset{n}{\underset{j=1}{\sum }}\frac{S_{ij}}{Z_{i}}\log\left(\frac{S_{ij}}{Z_{i}}\right),H_{j}^{+}=-\overset{n}{\underset{i=1}{\sum }}\frac{S_{ij}}{A_{j}}\log\left(\frac{S_{ij}}{A_{j}}\right), \end{array}$$

where $Z_{i}=\overset{n}{\underset{j=1}{\sum }}S_{ij},A_{j}=\overset{n}{\underset{i=1}{\sum }}S_{ij}$. Entropy is a measure of disorder, uncertainty, or surprise that has been extensively studied in physics and information theory. In this context, $H_{i}^{-}$ measures how much the *i*th topic from before *t* can be “explained away” by a single topic from after *t*. For example, the entropy is at its minimum, 0, when the similarity between the *i*th pre-*t* topic is non-zero for only one post-*t* topic; let's say with $T_{j}^{+}$. This is the case when every paper in both $c_{i}^{-}$ and $c_{j}^{+}$ belong to the same cluster *c*_*k*_, a compelling argument for a one-to-one matching between $c_{i}^{-}$ and $c_{j}^{+}$ that *did not depend* on any human comparison of keywords. Meanwhile, the maximum entropy (log*n*) is achieved when the *i*th pre-*t* topic is equally similar to all post-*t* topic vectors. We interpret this uncertainty to mean that the papers have spread out and the topic has potentially dissipated after *t*. On the other hand, if $H_{j}^{+}$ is high, then papers from all across the before topics came together to form the cluster $c_{j}^{+}$, which can be interpreted as the formulation of a new coherent topic after *t*. The “high entropy" cases could perhaps be interpreted as (mini-) paradigm shifts (Kuhn, 1970)–a disruption of tradition through the disintegration of an old sub-field or the formation of a new one–which more recent sociology of science suggests can occur at many scales and higher frequency (Foster et al., 2015).

## 3. Results

This section compares topics in cognitive science literature before and after 2012 based on the proposed similarity score and entropy measure, and identifies a few interesting topics that have changed. We present a few case studies by investigating these topics in the dimensions of keywords, affiliations, and journals.

### 3.1. Topic similarity

**Figure 1:**
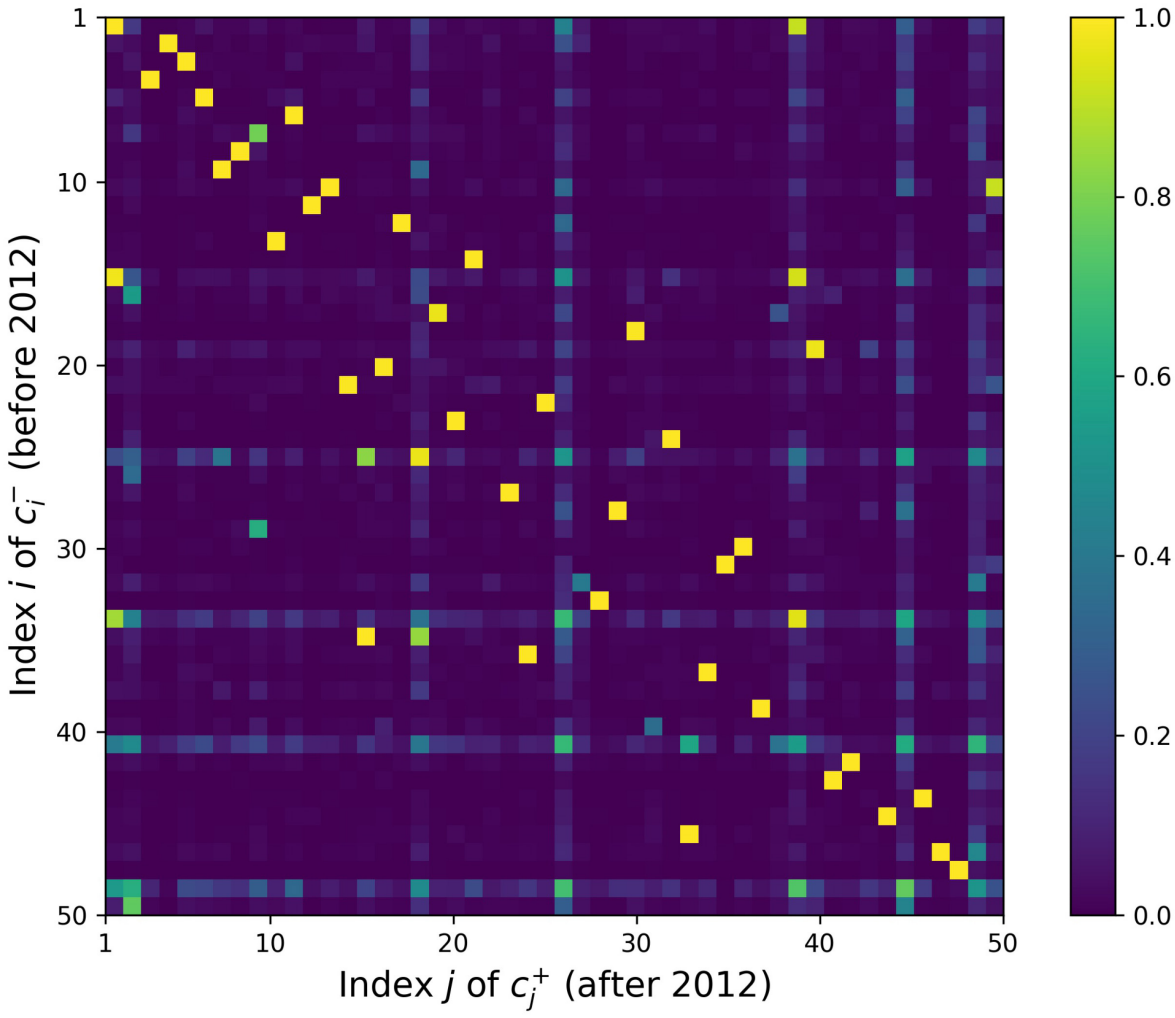
Topic similarity matrix *S*. The element *S*_*ij*_ in the *i*th row and *j*th column represents the similarity between the *i*th before topic and *j*th after topic in terms of paper distributions.

Figure 1 shows the heat map for the similarity matrix *S*. Many topics have a before-after match with cosine similarity close to 1, e.g., *S*_8, 8_ = 0.9997, where $c_{8}^{-}$ (947 papers) and $c_{8}^{+}$ (725 papers) share the top two keywords “memory” and “retrieval.” However, in many cases these strong matches correspond to off-diagonal elements. Recall that the topic clusters are in descending order; the indices correspond to the rank of the topic, as measured by the relative number of papers in each corpus. Then, when the ranks of the matching clusters differ, it may relate to a rise or decline in the importance of the topic in the research community. For example, $c_{21}^{-}$ (625 papers) and $c_{14}^{+}$ (573 papers) have top keywords such as “social,” “individual,” “social interaction,” “network,” “interaction,” and *S*_21, 14_ = 0.9932. Increase in social network analysis since 2012 may have pushed this topic from rank 21 to 14. On the other hand, $c_{18}^{-}$ (651 papers, keywords “knowledge,” “domain,” “acquire,” “expert,” “know”) and $c_{30}^{+}$ (468 papers, keywords “knowledge,” “conceptual,” “domain,” “logic,” and “world”) are also very similar at *S*_18, 30_ = 0.9959, but the ranking decreased from 18 to 30. This may correspond to a decreased interest in expertise, once a prominent topic in cognitive science, paralleling interest in expert systems and knowledge engineering in AI.

**Figure 2:**
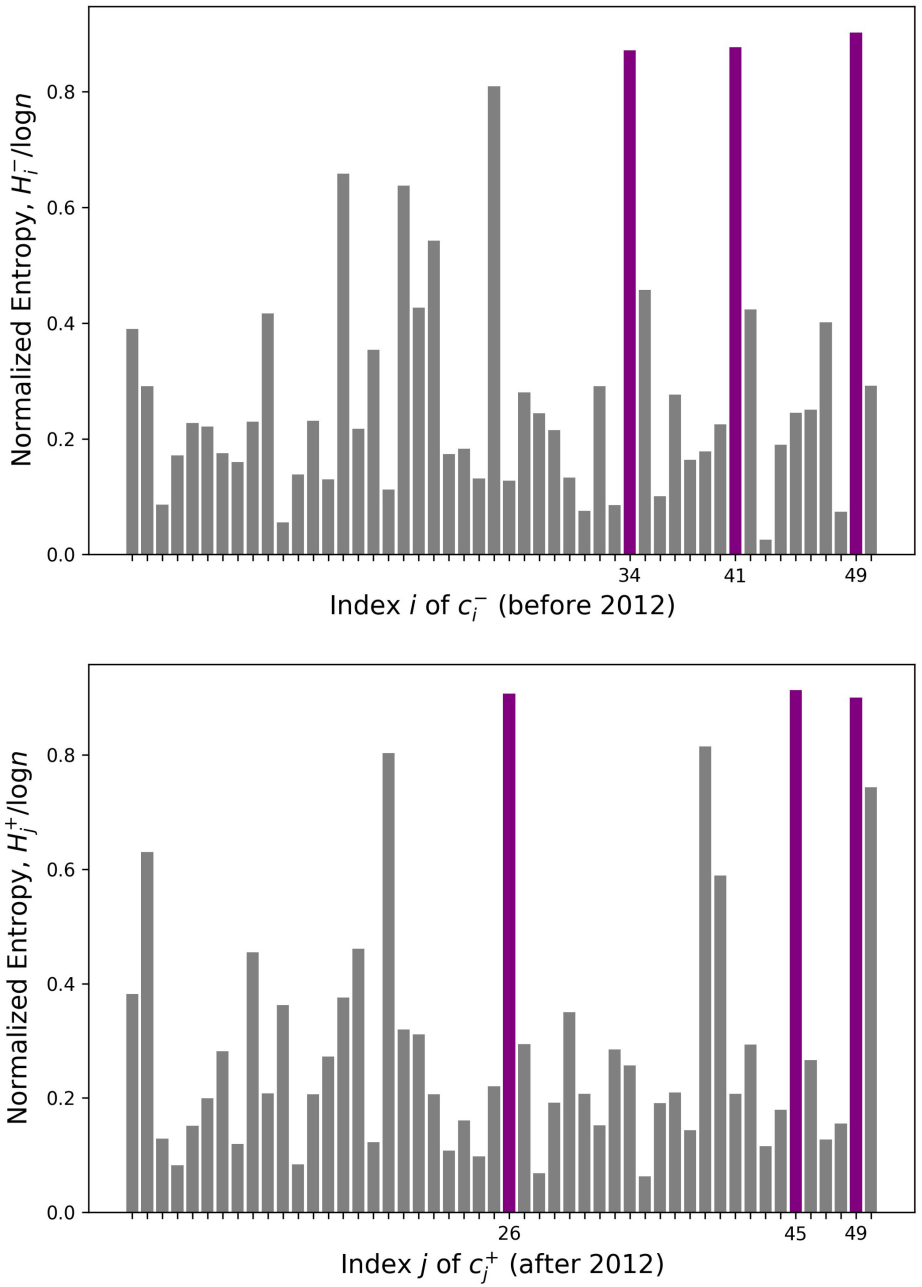
Entropy for every before and after topic. Three highlighted topics have the highest entropy.

However, a few topics such as $c_{41}^{-}$ have significant similarities with many other topics, making it hard to pinpoint a single good match. While these topics in question are recognizable by eye as blue-green horizontal and vertical lines in Figure 1, we quantify this uncertainty rigorously with entropy. The calculated entropy values are plotted in Figure 2, where the topics with three highest entropy are highlighted in purple. Table 1 lists the keywords attached to those topics of interest, which are potentially newly-formed or disappeared/dissipated topics. In the next section, we dig deeper into two particular cases to further examine how they have changed.

**Table 1:** Top keywords associated with the high entropy topics from Figure 2.

**Before 34**	**Before 41**	**Before 49**	**After 26**	**After 45**	**After 49**
question	time	paradigm	art	interaction	narrative
answer	event	symbolic	technology	context	story
address	change	ai	digital	dynamic	event
whether	temporal	connectionist	practice	environment	character
issue	dynamic	computational	aesthetic	embodied	generation
raise	state	classical	image	interactive	structure
ask	sequence	connectionism	cultural	individual	storytelling
how do	occur	methodology	Medium	activity	reader
think	past	within	culture	perspective	text
way	future	traditional	artistic	multimodal	world

Highlighted columns are discussed as case studies in Section 3.2.

### 3.2. Case studies: Before 49 and after 26

Based on the keywords in Table 1, the before 49 topic seems to be related to the symbolic AI vs. connectionist AI debate which was a hot topic in the 1960s–1990s (Smolensky, 1987). Symbolic AI was gradually abandoned starting in the 1990s due to issues discussed by philosopher Hubert Dreyfus (Dreyfus, 1992), and was put to bed once artificial neural networks (based on connectionist approaches to AI) rose to prominence. The top journals in Table 2 are *Trends in Cognitive Sciences* and *Minds and Machine*, which are two big journals in cognitive science, the latter focusing on the intersection of AI, cognitive science, and philosophy (an appropriate venue for such a debate). It further shows that the topic was quite promising at one point before it disappeared. We also note that almost none of the top keywords except “AI” appear as a top key word in any of the topics after 2012. The “symbolic/connectionist" topic seems to disappear after new deep learning methods demonstrated their practical efficacy, offering a pragmatic (and perhaps premature) conclusion to philosophical debates about the best approach to AI. AI remains a hot topic, of course, while previously pressing discussions of the major “paradigms” of “symbolic” AI and “connectionist” AI have faded into obscurity.

**Table 2:** Contextual information for before topic 49 (404 papers) and after topic 26 (474 papers).

	**Top 5 Affiliations**		**Top 5 Journals**	
Before 49	Indiana University	11	Trends in Cognitive Sciences	5
	University of California Berkeley	9	Minds and Machines	5
	Harvard University	8	Kybernetes	4
	University of Michigan	7	J. of Experimental Psychology General	3
	Complutense University of Madrid	7	J. of Educational Psychology	3
After 26	Johns Hopkins University	13	Leonardo	9
	University of Vienna	12	Behavioral and Brain Sciences	6
	University College London	10	Progress in Brain Research	6
	Shanghai University	8	Frontiers in Psychology	5
	University of California Los Angeles	7	AI & Society	4

The keywords in after topic 26 roughly talk about the intersection of “art” and “technology” and how they are related to “digital” “culture” and society. Table 2 shows the top affiliations and journals. Johns Hopkins University and University of Vienna are two leading institutions on this topic, with 13 and 12 papers in the topic affiliated. The top journal, *Leonardo*, is a journal published by MIT Press covering the applications of technologies to arts and music, which matches our interpretation of the keywords. As technology becomes more prominent and powerful in people's daily lives, discussions on art, technology and aesthetic practices have also increased–a trend we can only imagine will continue with the rise of Midjourney (Midjourney, 2022) and DALL-E 2 (Ramesh et al., 2021).

## 4. Discussion

In this paper, we propose a new unsupervised learning method to identify topic trends and shifts from a corpus of academic papers. We demonstrate it by exploring two sets of topics in cognitive science: one set that was once popular but disappeared or subdivided over time, and another that has emerged since the revival of neural networks in 2012. We provided a simple interpretation based on keywords, affiliations and journals. We also note that our topics are consistent with the account of Núñez et al. (2019), with most topics reflecting cognitive psychology quite prominently and topics related to anthropology or philosophy quite rare. We did, however, find neuroscience and CS/AI playing quite a prominent role, especially in post-2012 topics. We could explore the degree of integration across these constituent fields by looking at the co-appearance of related topics across documents: an excellent topic for further work. As a further check on our method, we also split the corpus into roughly equal chunks from 1950–1986 to 1987–1994, basing our checkpoint on the publication of the famous *Parallel Distributed Processing* book (Rumelhart et al., 1986). With this earlier split, more philosophical topics were prominent in *c*^−^, while new topics explicitly dealing with connectionism appeared in *c*^+^. We found further corroboration of our method in identifying dissipating topics like the language-training of apes, significantly disrupted by the work of Herb Terrace. Most intriguing, a pilot analysis of the entropy before and after 1986 suggests that (Rumelhart et al., 1986) was more “disruptive" of the topical content of cognitive science than (Krizhevsky et al., 2012); this makes sense, as the former occurred within the field and the latter adjacent to it.

When identifying the newly appeared and disappeared topics, we used the similarity matrix *S* based on using cosine similarity, which normalizes the vectors to obtain a value between 0 and 1. This can help us find two topics that are highly similar. Yet this metric could have two spread out topics appear almost identical when in fact they are completely different. It also does not take into account the actual number of papers in each topic, which could influence the size of the effect some topics have on others.

In the future, we could make use of more fine-tuned or model-based metrics to quantify the similarity between different set of topics; such metrics could incorporate not only shared number of papers, but also common keywords, authors and affiliations, etc. While we assigned each paper to only one topic at this stage, it is straightforward to assign “fractional” papers to topics based on the distributions. We could also employ causal inference techniques to identify potential causes for topics to appear/disappear or become popular/divide; likewise we can find pivotal change-points by finding especially high-entropy splits and then try to identify the causal drivers of such change. Even within the specific field of cognitive science, there is considerable space for in-depth discussions of newly formed topics— both their content and their socio-institutional context (e.g., using our method to guide a more traditional history of science approach). Finally, we can use prediction models to predict whether a topic is likely to appear or disappear in the future; this would be hugely beneficial to funders and practicing scientists alike.

## Data availability statement

Publicly available datasets were analyzed in this study. This data can be found at: https://github.com/HarlinLee/cogsci-missed-connections.

## Author contributions

LC, HL, and JF contributed to the conception and design of the study and revised the manuscript. LC and HL performed the data analysis. LC wrote the first draft of the manuscript. All authors contributed to the article and approved the submitted version.

## Funding

This work was supported by grant TWCF0333 from the Templeton World Charity Foundation.

## Conflict of interest

The authors declare that the research was conducted in the absence of any commercial or financial relationships that could be construed as a potential conflict of interest.

## Publisher's note

All claims expressed in this article are solely those of the authors and do not necessarily represent those of their affiliated organizations, or those of the publisher, the editors and the reviewers. Any product that may be evaluated in this article, or claim that may be made by its manufacturer, is not guaranteed or endorsed by the publisher.

## Footnotes

^1^We get a similar result using kurtosis, which is another measure of concentration.
