# Supplementary material for: A Simple, interpretable method to identify surprising topic shifts in scientific fields
Source: Front Res Metr Anal. 2022 Oct 12;7:1001754. doi: 10.3389/frma.2022.1001754 (PMC9597295; doi:10.3389/frma.2022.1001754)
Supplement: Supplementary file 1 [file Data_Sheet_1.PDF]

Supplementary Material

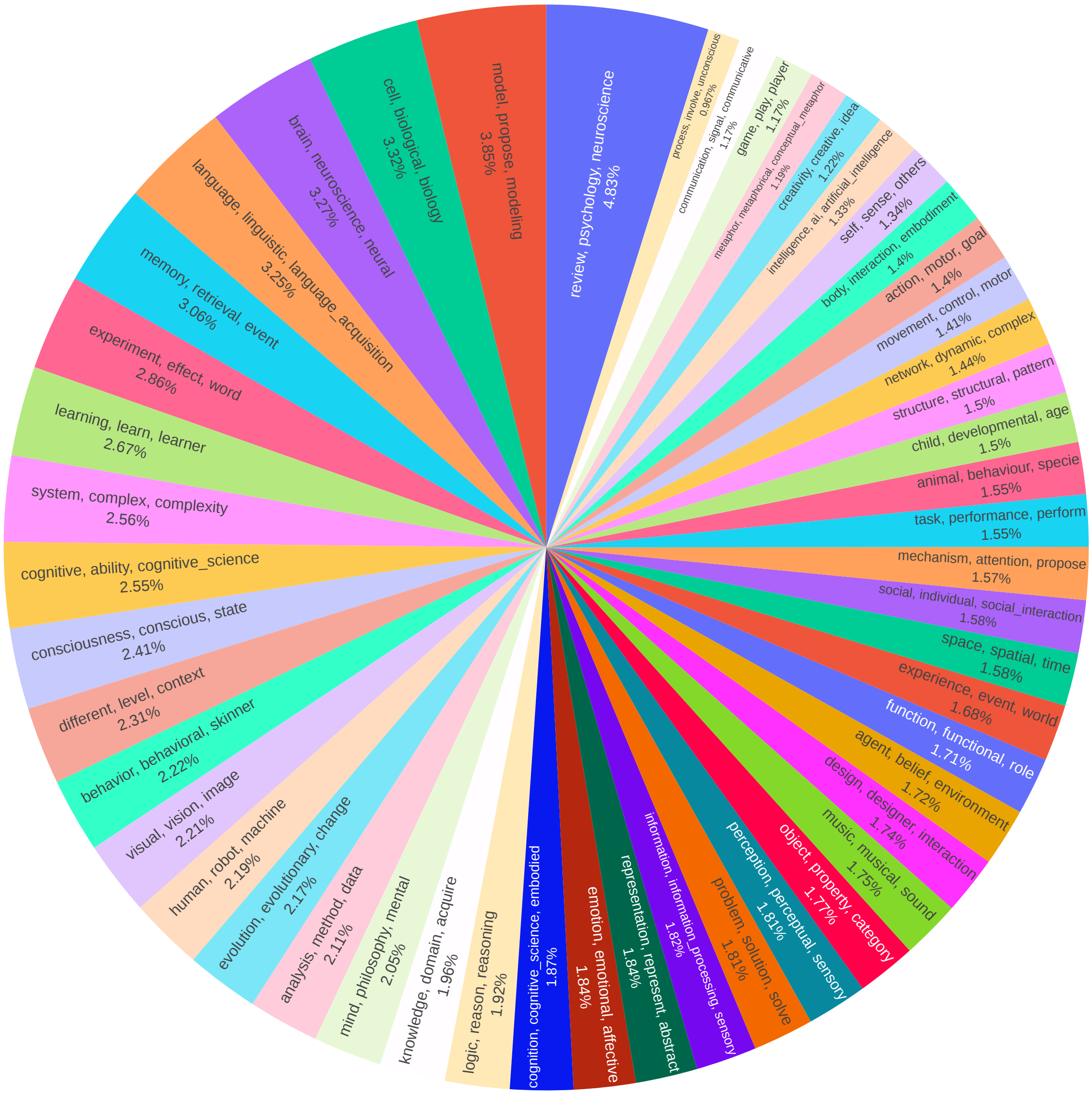

Figure S1. Topic clusters from overall text corpus.

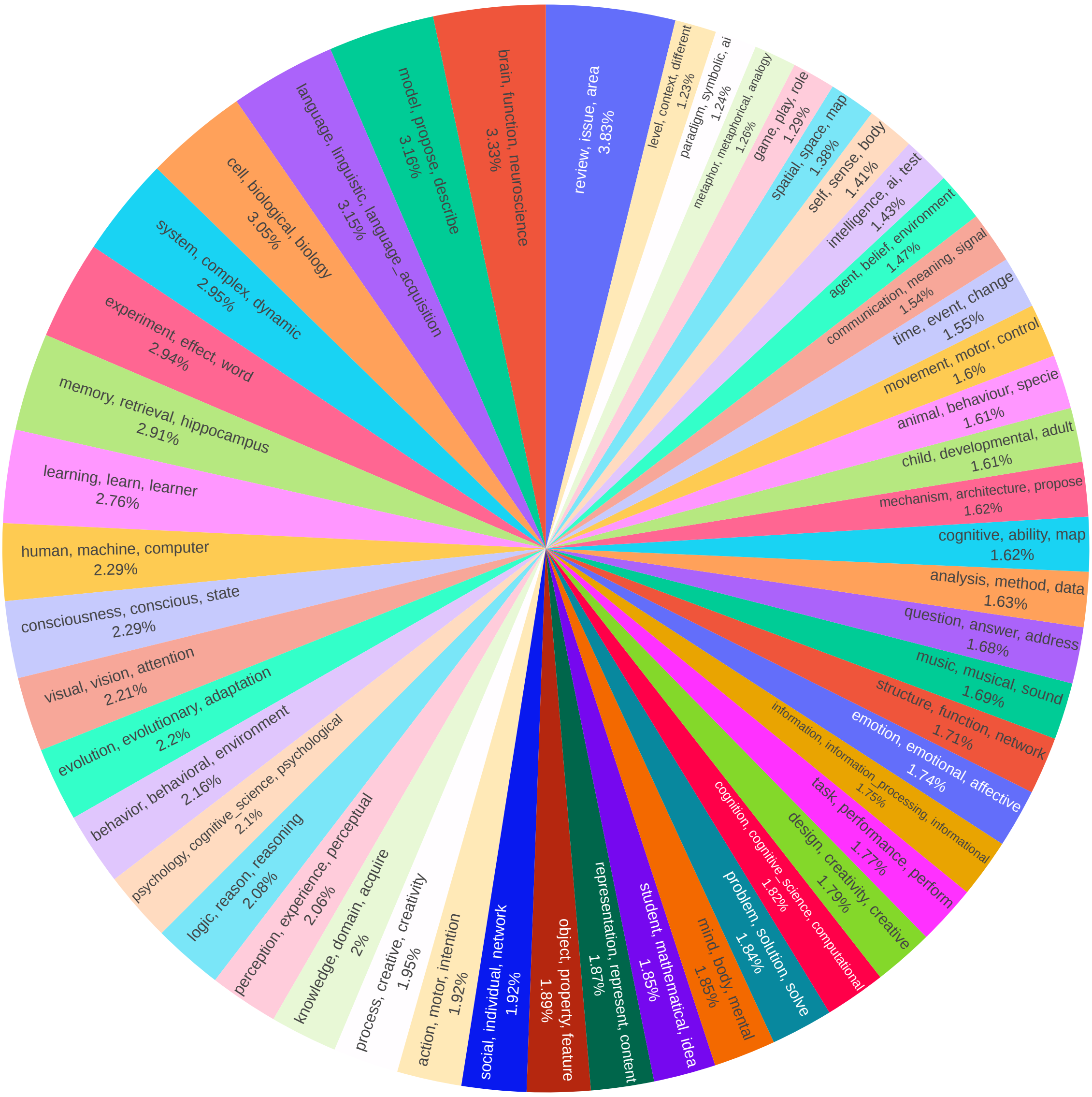

Figure S2. Topics before 2012

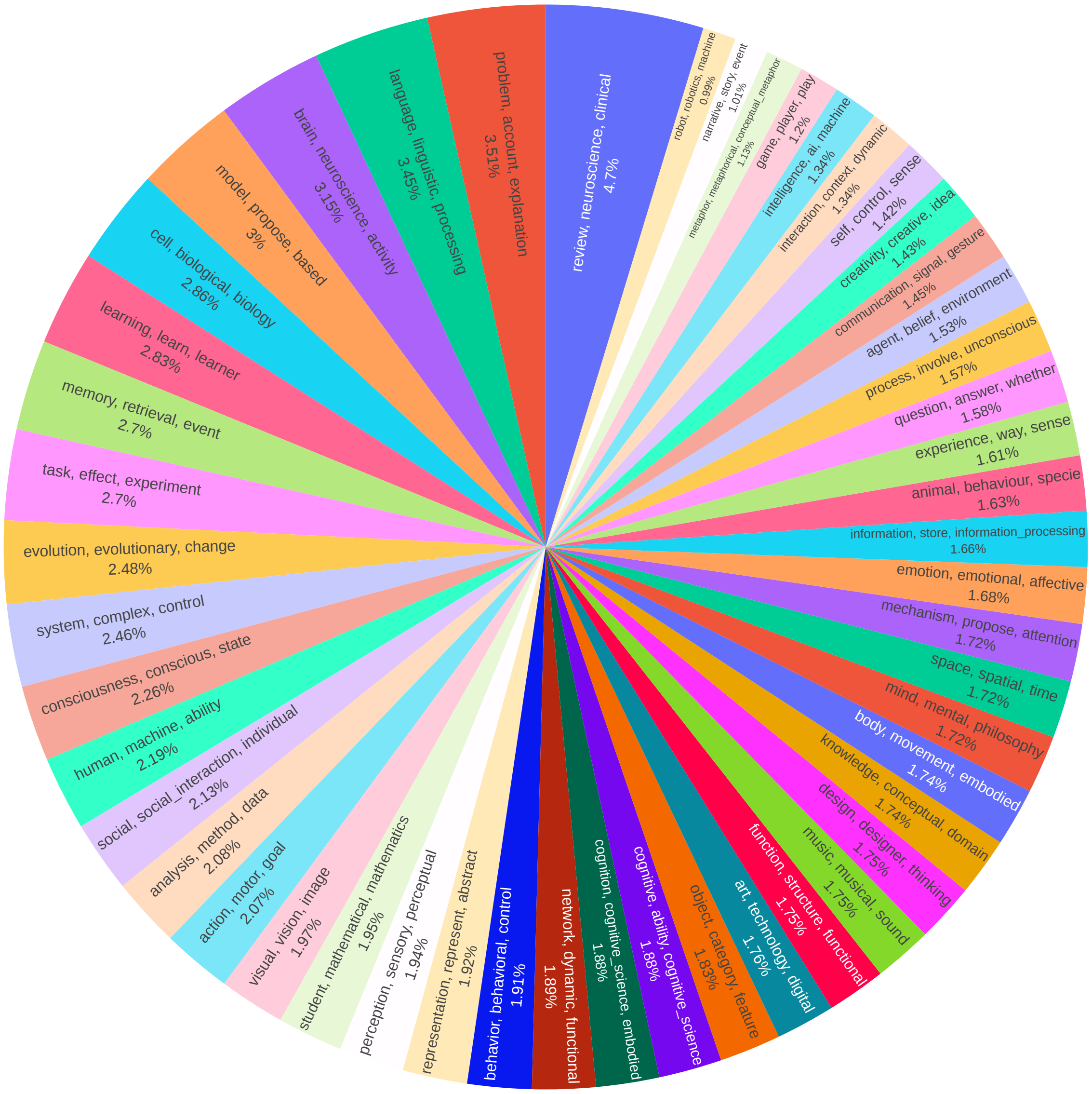

Figure S3. Topics after 2012.
